# Increased Tea Consumption Is Associated with Decreased Arterial Stiffness in a Chinese Population

**DOI:** 10.1371/journal.pone.0086022

**Published:** 2014-01-22

**Authors:** Chung-Hao Li, Yi-Ching Yang, Jin-Shang Wu, Ying-Hsiang Huang, Chih-Ting Lee, Feng-Hwa Lu, Chih-Jen Chang

**Affiliations:** 1 Department of Family Medicine, Tainan Municipal An-Nan Hospital- China Medical University, Taiwan; 2 Department of Family Medicine, National Cheng Kung University Hospital, Tainan, Taiwan; 3 Department of Family Medicine, College of Medicine, National Cheng Kung University, Tainan, Taiwan; University of Perugia, Italy

## Abstract

**Background:**

Tea has attracted considerable attention for its potential cardioprotective effects. The primary chemical components of tea are thought to have a beneficial effect by reducing arterial stiffness. The objective of this study was to assess the association between tea consumption and brachial–ankle pulse wave velocity (baPWV) in a relatively healthy Chinese population.

**Methods:**

We enrolled 3,135 apparently healthy subjects from October 2006 to August 2009. Subjects taking medication for diabetes, hypertension, or hyperlipidemia, or with a history of cardiovascular disease, were excluded from the study. The subjects were categorized into three groups according to their tea-drinking habits: (1) none to low (n = 1615), defined as non-habitual tea drinkers, or drinking for <1 year, or drinking ≤150 mL per day for ≥1 year ; (2) moderate tea consumption, defined as drinking for ≥1 year and consumption between 151 and 450 mL per day; and (3) heavy tea consumption, defined as a drinking for ≥1 year and consumption >450 mL per day. Multiple logistic regression was used to determine whether different levels of consumption were independently associated with the highest quartile of baPWV values, defined as ≥1428.5 cm/s.

**Results:**

Of the 3,135 subjects, 48.5% had drunk >150 mL of tea per day for at least 1 year. In multivariate regression analysis with adjustment for co-variables, including, age, sex, current smoking, alcohol use, habitual exercise, total cholesterol/high-density lipoprotein cholesterol (TC/HDL-C) ratio >5, obesity, newly diagnosed hypertension and diabetes, subjects with high tea consumption had a decreased risk of highest quartile of baPWV by 22% (odds ratio = 0.78, 95% confidence interval = 0.62–0.98, *p* = 0.032), while subjects with moderate tea consumption did not (*p* = 0.742), as compared subjects with none to low tea consumption.

**Conclusions:**

High, but not moderate, habitual tea consumption may decrease arterial stiffness.

## Introduction

Arterial stiffness is characterized by arterial wall thickening and loss of elasticity, and reflects a reduction in vascular compliance involving structural and cellular elements of the vessel wall [1]. Independent of traditional cardiovascular risk factors, arterial stiffness is a predictor of total mortality and future cardiovascular disease, such as myocardial infarction, heart failure, and stroke [1], [2]. Pulse wave velocity (PWV) is the most validated of the non-invasive methods used to assess arterial stiffness [3]. The gold standard for assessment is measurement of central arterial stiffness via the carotid–femoral PWV (cfPWV), although this is inconvenient due to the requirement of exposure of the inguinal region [4]. In contrast, brachial–ankle PWV (baPWV) requires only a pressure cuff to be wrapped around both arms and ankles, and can be reproducibly measured. Recent reports have also shown that baPWV strongly correlates with carotid–femoral PWV, which has resulted in wide use of the technique in a majority of Asian countries [4].

Evidence on strategies involving lifestyle issues is still required to obtain a better understanding of how to reduce arterial stiffening [5]. Tea, the most commonly consumed beverage after water, has attracted considerable attention for its potential cardioprotective effects [6]. Polyphenols are the primary chemical components of tea responsible for the beneficial effects on cardiovascular disease [7], and flavonoids, which are the main polyphenols present in tea, have been elucidated to be related to the vascular health [8]. Therefore, polyphenols may also be beneficial in reducing arterial stiffness. One cross-sectional study found no relationship between green tea drinking and arterial stiffness in patients with type 2 diabetes, although the sample size of this study was relatively small [9]. Larger prospective studies to determine the precise causality between tea consumption and baPWV measurements are currently unavailable. The objective of the present study in a Chinese population without known hypertension, diabetes, and cardiovascular diseases was therefore to carry out a detailed assessment of the association between different status of tea consumption and baPWV, taking into account potential confounding factors of arterial stiffness.

## Methods

### Subjects and data collection

Subjects who had a physical check-up at the health examination center of the National Cheng Kung University Hospital between October 2006 and August 2009 were enrolled in the study. Each individual completed a structured questionnaire, which recorded demographic information, lifestyle habits, and medical and medication history. The study exclusion criteria included a history of hypertension, diabetes mellitus, coronary heart disease, old stroke, anemia (hemoglobin: men, <13.5 g/dL; women, <12.0 g/dL), amputation of either lower limbs, alcohol drinking >30 g per week, taking medications known to influence BP, plasma glucose and lipid profile, and incomplete questionnaire information. Subjects with peripheral atherosclerosis and an ankle–brachial index (ABI) <0.95 were also excluded to ensure accurate measurement of baPWV [10]. A cross-sectional sample of 3,135 consecutive subjects (men, 60.4%; women, 39.6%) who met these criteria was included in the final analysis using secondary data without personal identification information. Written consent was obtained from all participants, and the study was approved by the Ethical Committee for Human Research at National Cheng Kung University Hospital, Taiwan.

All measurements were performed using standard methods by well-trained nurses. Anthropometric measurements were obtained with each subject in light clothing and without shoes. Body height to the nearest 0.1 cm and weight to the nearest 0.1 kg were measured using a certified machine, and body mass index (BMI) calculated and expressed as weight in kilograms divided by the height in meters squared. Supine readings of brachial systolic and diastolic blood pressure (SBP, DBP) were measured after at least 15 min rest using a DINAMAP TM vital signs monitor (Model 1846SX, Critikon Inc, Irvine, California, U.S.A.). Mean SBP and DBP were calculated by averaging the right and left BP readings.

All subjects had blood samples collected after an overnight fast of at least 8 h for measurement of fasting plasma glucose (FPG), total cholesterol (TC), triglyceride, and high-density lipoprotein cholesterol (HDL-C) concentrations. Two-hour post load glucose (2-h PG) levels were measured after a 75-g oral glucose tolerance test in non-pregnant women and subjects without a history of diabetes. FPG and 2-h PG levels were determined using the glucose oxidase method (Synchron CX3, Beckman Coulter Inc., Brea, California, U.S.A). Lipid profiles were enzymatically determined (Roche Diagnostics, Mannheim, Germany) using a clinical chemistry analyzer (Roche Modular DP).

### Assessment of tea consumption

A tea consumption questionnaire has been used in other research [11], which was used as the basis for the one used in this work. The first question for assessing tea consumption was “Have you drunk tea habitually once a week for at least 6 months?” Subjects who answered “yes” then completed the following questions and were coded as habitual drinkers; otherwise, they were recorded as non-habitual drinkers. 1) How many times do you drink tea each day? 2) What kind of cup was used each time? For example, 30 mL per cup for Chinese so-called “elderly tea,” 150 mL per cup for a typical afternoon tea in a Western restaurant, 250 mL for tea drunk in a mug, 300 mL per pack for aluminum foil packed tea, and 350 mL per can for canned tea. Note that we provided these examples in the questionnaire to assist the subjects to help them answer the following question more accurately. 3) How much (milliliters) tea do you drink each day? 4) How many years have you been drinking tea in this way?

Using this self-administered dietary questionnaire, we categorized the subjects into three subgroups: 1) none or low tea consumption, defined as non-habitual tea drinkers, or drinking tea for less than one year, or drinking tea for at least one year with consumption ≤150 mL per day; 2) moderate tea consumption, defined as drinking tea for at least one year with consumption of between 151 and 450 mL per day; 3) heavy tea consumption, defined as drinking tea for at least one year with consumption of >450 mL per day.

### Pulse wave velocity

The baPWV values, calculated as the distance traveled by the pulse wave divided by the time taken to travel the distance, were assessed using an automatically non-invasive vascular screening device (BP-203RPE II; Colin Medical Technology, Komaki, Japan). This was achieved by wrapping four pneumatic pressure cuffs around each of the four extremities that simultaneously measured the blood pressure levels and pulse waves of the brachial artery of both arms and tibial artery of both legs after 5 min of bed rest [4]. Although the baPWV values were bilaterally measured, the mean baPWV value was used due to a significant positive correlation between the left and right baPWV (r = 0.975, *p* value<0.001). The mean baPWV was also divided into quartiles: Q1, ≤1169.0; Q2, 1169.5–1282.5; Q3, 1283.0–1428.0; Q4, ≥1428.5 cm/s.

### Definitions

Newly diagnosed hypertension was defined as a mean SBP>140 mm Hg and/or mean DBP>90 mm Hg [12], without a history of hypertension. Newly diagnosed diabetes was defined as a FPG≥7.0 mmol/L or 2-h PG≥11.1 mmol/L [13], without a history of diabetes. Obesity was defined as BMI≥25 kg/m^2^, based on the World Health Organization's Asia Pacific guidelines. The total cholesterol (TC)/HDL-C ratio was calculated and categorized as either ≤ 5.0 or >5.0. Smoking and alcohol consumption status was assessed by asking each participant whether they were a current smoker or alcohol drinker. Habitual exercise was categorized as vigorous exercise at least three times weekly [14].

### Statistical analyses

The data were analyzed using SPSS for Windows (version 17.0; SPSS. Chicago, IL, USA). The level of tea consumption was expressed as median and inter-quartile ranges, as the data had a non-parametric distribution. The other data were expressed as mean ± standard deviations for continuous variables (except mean ± standard error for baPWV values) or as number and percentage for categorical variables. Comparisons of clinical characteristics between subgroups according to quartiles of baPWV were carried out using Pearson chi-square tests for categorical data or one-way ANOVA for continuous data. The Kruskal-Wallis test was used for comparisons of different levels of tea consumption. Across group differences detected in baPWV were analyzed further using Scheffé's post-hoc pairwise test. Multiple logistic regression was used to determine whether different levels of tea consumption were associated independently with the highest quintile of baPWV (Q4, dependent variable). Other independent variables included age (<40, 40–60, >60 years), sex, TC/HDL-C ratio >5.0, current smoker, current alcohol drinker, habitual exercise, and newly diagnosed hypertension, diabetes, or obesity. The odds ratios (OR) and 95% confidence intervals (CIs) of the predictors were derived for the regression model. Statistical significance was defined as a *p* value<0.05.

## Results

Table 1 shows the baseline characteristics of the study subjects grouped according to quartiles of baPWV. There were significant differences in sex, age, BMI, SBP, DBP, FPG, 2-h PG, TC, triglyceride, HDL-C, daily tea consumption, and proportions of current alcohol drinkers and smokers in the four groups. There was a positive linear trend for the association between different quartiles of baPWV and the prevalence of TC/HDL-C ratio >5.0, obesity, and newly diagnosed hypertension, and diabetes.

**Table 1:** Comparison of clinical characteristics among study subjects grouped according to quartiles of baPWV.

Characteristic	Quartiles of baPWV*				*p* value
	Q1	Q2	Q3	Q4	
	(n = 783)	(n = 786)	(n = 781)	(n = 785)	
Male sex	321 (410)	486 (61.8)	562 (72.0)	524 (66.8)	<0.001§
Age, years	38.1±8.9	43.3±9.2	47.1±9.2	55.5±11.1	<0.001†
Body mass index, kg/m^2^	22.9±3.6	23.7±3.4	24.4±3.4	24.6±3.2	<0.001†
Systolic blood pressure, mmHg	105.2±9.1	112.1±9.6	119.1±11.2	130.5±15.4	<0.001†
Diastolic blood pressure, mmHg	60.9±6.9	66.9±8.0	72.6±8.9	78.3±10.5	<0.001†
FPG, mmol/l	4.70±0.71	4.81±0.76	4.97±1.02	5.14±1.13	<0.001†
2-h PG, mmol/l	5.49±1.73	6.01±2.17	6.44±2.75	7.23±3.04	<0.001†
Cholesterol, mmol/l	4.82±0.90	5.03±0.93	5.18±0.90	5.29±0.96	<0.001†
Triglyceride, mmol/l	1.12±0.75	1.31±0.82	1.52±1.13	1.49±0.90	<0.001†
HDL-C, mmol/l	1.43±0.39	1.34±0.35	1.28±0.34	1.31±0.35	<0.001†
Tea consumption, mL/day¶	200 (0–500)	200 (0–500)	240 (0–500)	120 (0–500)	0.003‡
Current alcohol drinker	92 (11.7)	139 (17.7)	174 (22.3)	129 (16.4)	<0.001§
Current smoker	91 (11.6)	121 (15.4)	134 (17.2)	117 (14.9)	0.019§
Habitual exercise (≥3 times/week)	13 (1.7)	21 (2.7)	20 (2.6)	26 (3.3)	0.224§
TC/HDL-C>5	90 (11.5)	144 (18.3)	198 (25.4)	214 (27.3)	<0.001§
Obesity	184 (23.5)	230 (29.3)	312 (39.9)	328 (41.8)	<0.001§
Newly diagnosed hypertension	0 (0.0)	6 (0.8)	51 (6.5)	206 (26.2)	<0.001§
Newly diagnosed diabetes	6 (0.8)	17 (2.2)	34 (4.4)	66 (8.4)	<0.001§

Abbreviation: FPG, fasting plasma glucose; 2-h PG, two-hour post load glucose; TC, total cholesterol; HDL- C, high–density lipoprotein cholesterol; baPWV, brachial–ankle pulse wave velocity.

Data are expressed as mean ± SD or number (percentage).

¶Data are expressed as median (inter-quartile ranges).

*Quartiles of baPWV: Q1, ≤1169.0; Q2, 1169.5–1282.5; Q3, 1283.0–1428.0; Q4, ≥1428.5 cm/s.

†*p*<0.05, for ANOVA.

‡*p*<0.05, for Kruskal–Wallis test.

§*p*<0.05, for Pearson chi-square test.

**Figure 1:**
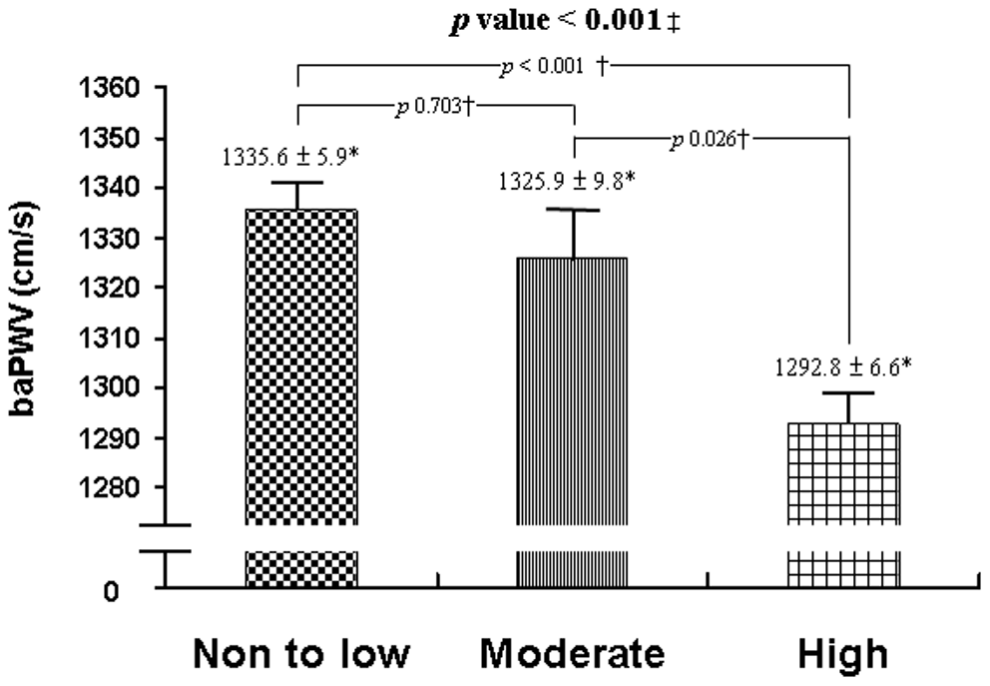
Comparison of baPWV in subjects with no to low, moderate, or high tea consumption. ‡ANOVA among groups; † Scheffé's post-hoc test; *Data are expressed as mean ± standard error.

Of the 3,135 subjects, 48.5% had drunk >150 mL of tea each day for at least one year. Table 2 shows the data of the subjects grouped according to tea consumption: none to low (n = 1615), moderate (n = 507) and high (n = 1013). There were significant differences among the three groups for sex, age, BMI, DBP, triglyceride, HDL-C, proportion of TC/HDL-C ratio >5.0, obesity, and current alcohol drinkers and smokers. Comparisons of the mean values of baPWV among these three groups are shown in **Figure 1**, and there was also a significant difference in mean baPWV among the three groups (ANOVA, *p*<0.001). Scheffé's post-hoc test showed that subjects with high tea consumption had significantly lower baPWV values than those with none to low (*p*<0.001) and moderate consumption (*p = 0.026*), respectively. However, there was no significant difference in baPWV values between subjects with none to low and moderate tea consumption (*p* = 0.703).

**Table 2:** Comparison of clinical characteristics among study subjects grouped according to tea consumption.

Characteristic	Status of tea consumption*			*p* value
	None or low	Moderate	High	
	(n = 1615)	(n = 507)	(n = 1013)	
Male sex	924 (57.2)	331 (65.3)	638 (63.0)	0.001§
Age, years	47.4±12.0	45.9±10.9	43.8±10.8	<0.001†
Body mass index, kg/m^2^	23.6±3.4	24.2±3.2	24.3±3.6	<0.001†
Systolic blood pressure, mmHg	116.8±15.3	117.6±14.7	116.1±14.4	0.188†
Diastolic blood pressure, mmHg	69.3±10.8	70.8±10.8	69.6±10.8	0.031†
FPG, mmol/l	4.90±0.87	4.91±0.96	4.90±1.02	0.967†
2-h PG, mmol/l	6.26±2.43	6.50±2.74	6.24±2.65	0.127†
Cholesterol, mmol/l	5.08±0.93	5.11±0.95	5.07±0.94	0.694†
Triglyceride, mmol/l	1.32±0.91	1.41±0.97	1.40±0.92	0.026†
HDL-C, mmol/l	1.37±0.37	1.33±0.36	1.31±0.36	<0.001†
Current alcohol drinker	192 (11.9)	122 (24.1)	220 (21.7)	<0.001§
Current smoker	168 (10.4)	92 (18.1)	203 (20.0)	<0.001§
Habitual exercise (≥3 times/week)	40 (2.5)	10 (2.0)	30 (3.0)	0.121§
TC/HDL-C>5	298 (18.5)	106 (20.9)	242 (23.9)	0.004§
Obesity	488 (30.2)	179 (35.3)	387 (38.2)	<0.001§
Newly diagnosed hypertension	134 (8.3)	44 (8.7)	85 (8.4)	0.964§
Newly diagnosed diabetes	62 (3.8)	23 (4.5)	38 (3.8)	0.735§

Abbreviation: FPG, fasting plasma glucose; 2-h PG, two-hour post load glucose; TC, total cholesterol; HDL-C, high-density lipoprotein cholesterol; baPWV, brachial–ankle pulse wave velocity.

Data are expressed as mean ± SD or number (percentage).

Status of tea consumption: 1) None or low, defined as non-habitual tea drinkers, a drinking habit less than one year or a daily tea drinking amount ≤150 mL per day for at least one year; 2) Moderate, defined as daily tea consumption between 151 and 450 mL per day for at least one year; 3) High, tea consumption >450 mL per day for at least one year.

†*p*<0.05, for ANOVA.

§*p*<0.05, for Pearson chi-square test.

The results of the multiple logistic regression analyses are summarized in Table 3. After adjustment for the covariables, including age, sex, current smoker, current alcohol drinker, habitual exercise, TC/HDL-C ratio>5.0, obesity, newly diagnosed hypertension and diabetes, subjects with high tea consumption had a 22% decreased risk of being in the highest quartile of baPWV (*p* = 0.032) compared with subjects with none to low tea consumption. But subjects with moderate tea consumption did not show this significant reduction (*p* = 0.742). In addition, age, TC/HDL-C ratio>5.0, newly diagnosed hypertension and newly diagnosed diabetes were independently associated with the risk of being in the highest quartile of baPWV.

**Table 3:** Multiple logistic regression model for the relationship between tea consumption and highest quartile of baPWV.

Independent variables	odds ratio (95% confidence interval)	*p* value
Age, years		
40–60 vs. <40	4.41 (3.28–5.92)	<0.001
>60 vs. <40	35.69 (24.52–51.96)	<0.001
Sex, female vs. male	0.88 (0.70–1.09)	0.247
Tea consumption, mL/day		
moderate vs. none or low	0.96 (0.73–1.26)	0.742
high vs. none or low	0.78 (0.62–0.98)	0.032
Current smoker, yes vs. no	1.16 (0.87–1.55)	0.307
Current alcohol drinker, yes vs. no	0.83 (0.63–1.09)	0.177
Habitual exercise, ≥3times/week vs.<3times/week	1.02 (0.57–1.83)	0.958
TC/HDL-C, >5 vs. ≤5	1.41 (1.11–1.79)	0.005
Obesity, yes vs. no	1.16 (0.94–1.43)	0.173
Newly diagnosed diabetes, yes vs. no	1.81 (1.16–2.82)	0.009
Newly diagnosed hypertension, yes vs. no	12.06 (8.63–16.86)	<0.001

Dependent variable: the highest quartile of baPWV (Q4, ≥1428.5 cm/s).

Abbreviation: TC, total cholesterol; HDL- C, high-density lipoprotein cholesterol; baPWV, brachial–ankle pulse wave velocity.

## Discussion

To the best of our knowledge, this is the first study to examine the relationship between arterial stiffness and tea consumption using detailed information adjusted for confounding effects. Our results showed that tea consumption greater than 450 mL/d for at least one year was inversely associated with the highest quartile of baPWV (≥1428.5 cm/s), independent of other cardiovascular risk factors, including, age, sex, lifestyle habits, TC/HDL-C ratio>5.0, obesity, and newly-diagnosed hypertension and diabetes. The cut-off point for the highest quartile of baPWV of ≥1428.5 cm/s used in the study may be clinically significant, as baPWV>1400 cm/s has been shown to be an independent risk factor for stroke or coronary heart disease [15]. Previously, only one study has investigated the effects of green tea on baPWV [9], and it focused on patients with type 2 DM. Their results showed no significant improvement in baPWV, possibly as a consequence of the relatively small sample size (n = 55). In contrast, our study showed a significant relationship between high tea consumption and decreased baPWV in a large sample of relatively healthy adults without a history of cardiometabolic diseases, even after adjustment for many cardiovascular risk factors.

What is the possible mechanism that links tea consumption and arterial stiffening? The benefits of tea consumption in cardiovascular health are thought to be due largely to flavonoids [7], [8], which are known to activate endothelial nitric oxide synthase (eNOS) [16]. Animal studies have shown that nitric oxide production regulates local arterial distensibility by modifying vascular smooth muscle cell tone [17]. It is therefore possible that tea consumption may reduce arterial stiffness by augmenting nitric oxide production [16]. Grassi D et al further showed dose-dependent effects of flavonoids improve endothelial function and increase peripheral arterial distensibility [18]. Moreover, flavonoids have been shown to have anti-oxidative and anti-inflammatory activities [6], and to activate macrophages, thereby protecting against the development of atherosclerosis [19]. Inhibition of atheroma formation, in turn, may decrease arterial stiffness by altering the mechanical properties of the arterial wall [20]. In addition, the beneficial effects of tea on cardiovascular disease risk factors, such as hypertension, dyslipidemia, diabetes, and obesity [6], may underlie the improvement of arterial stiffness observed in habitual tea drinkers.

Consistence with previous research, our findings also confirmed the detrimental effects of age, newly diagnosed hypertension and diabetes on arterial stiffness [21], [22]. Otherwise, we showed increased arterial stiffness was associated with a cut-point of TC/HDL-C ratio>5, a concordantly predictive of ischemic or congestive heart disease risk [23], [24]. Although the relationship between obesity, classified by BMI, and arterial stiffness was insignificant in our study, this finding was similar to the results reported by Miyai [25] and may be due to co-linearity after inclusion and adjustment for closely related variables, such as raised BP, dyslipidemia, and glucose intolerance.

Life style habites, such as smoking, alcohol drinking and exercise, was not associated with the risk of increased arterial stiffness after multi-factor adjustment in our study. The insignificant finding with regard to smoking may be due to misclassification of smoking status. Because subjects who had quit smoking would not be categorized as current smokers using our method of categorization, the residual effects of previous smoking on arterial stiffness are ignored [26]. In addition, current smokers may claim to have quit smoking, while still continuing to do so. As for exercise, previous evidence indicated that differences in exercise style (endurance or resistance) and period of exercising habitually (short- or long-term) may lead to inconsistent results, thereby explaining any discrepancies with earlier studies [5]. Regarding the effect of alcohol drinking on arterial stiffness, the Rotterdam study [27] found moderate alcohol consumption in women was associated with lower arterial stiffness, resulting in a U-shaped association. However, a nine-year longitudinal study carried out by Nakanishi *et al.* showed that PWV increased significantly in subjects who consumed more than 23 g of alcohol daily [28]. In our study, subjects consuming less than 30 g of alcohol per week showed an insignificant association between alcohol use and arterial stiffness. More studies are needed on the effect of alcohol use on arterial stiffness.

This study has certain limitations. First, it had a cross-sectional design, which did not enable us to draw any conclusions of a causal relationship between decreased arterial stiffness and habitual tea consumption. Second, although we attempted to minimize confounding effect by strict exclusion criteria and adjustment for major confounders in the final analysis, the presence of residual or unknown confounders cannot be ignored. In order to lessen the residual confounding effect of age, the age strata were further classified into four groups as <35, 35–50, 50–65, >65 year-old, respectively, and the result still showed a significant relationship between high tea consumption and decreased baPWV. Although 24 h sodium and potassium excretion were not available in our study, salt loading was highly correlated with blood pressure [29]. Similarly there was a significant association between insulin resistance and diabetes [30]. In multivariate analysis, we had adjusted for hypertension and diabetes, which might partially eliminate the confounding effect of 24 h sodium/potassium excretion and insulin resistance on the relationship between tea consumption and arterial stiffness. In addition, no diet history, especially flavonoid-rich food, was collected, such as a diary for daily diet intake recordings. The additive effect of other dietary flavonoids associated with tea drinking behavior on arterial stiffness was not completely ruled out in this study. Further studies are therefore required to clarify the independent effects of tea consumption and other flavonoid-rich food on arterial stiffness. Third, information on tea consumption may have been misclassified, despite the collection of detailed data, including both average daily consumption and the duration of this habitual use. Fourth, the kind of tea consumed was not grouped individually, as there is no clear evidence to date that different types of tea have superior cardiovascular effects [7]. Fifth, we used baPWV, instead of cfPWV which are the gold standard method, to measure arterial stiffness. This may suffer from some random error. However, baPWV is strongly correlated to cfPWV [4] and more widely used due to its reproducibility and simplicity. Thus, it may be reasonable to use baPWV for evaluation of arterial stiffness in this study. Finally, the study subjects were confined to a Chinese population, and therefore it remains to be established whether these results can be generalized to other ethnicities.

In conclusion, after adjustment for possible confounding factors, such as age, sex, current smoking, alcohol use, habitual exercise, total cholesterol/high-density lipoprotein cholesterol (TC/HDL-C) ratio>5, obesity, newly diagnosed hypertension and diabetes, our data indicates that the duration and amount of tea consumed are independently associated with arterial stiffness. More specifically, our study shows that habitual tea drinkers in a relatively healthy Chinese population without a history of cardiometabolic diseases who maintained a high level of tea consumption of more than 450 mL/d for at least one year had a decreased risk of abnormal arterial stiffness.
